# Reply to Fabbris et al. A Viable Alternative. Comment on “Kohmer et al. Self-Collected Samples to Detect SARS-CoV-2: Direct Comparison of Saliva, Tongue Swab, Nasal Swab, Chewed Cotton Pads and Gargle Lavage. *J. Clin. Med.* 2021, *10*, 5751”

**DOI:** 10.3390/jcm11164920

**Published:** 2022-08-22

**Authors:** Sebastian Hoehl, Niko Kohmer, Lisa Eckermann, Rene Gottschalk, Sandra Ciesek

**Affiliations:** 1 Institute for Medical Virology, University Hospital, Goethe University Frankfurt, 60596 Frankfurt, Germany; 2 Health Protection Authority, City of Frankfurt, 60313 Frankfurt, Germany; 3 German Centre for Infection Research, External Partner Site, 60323 Frankfurt, Germany; 4 Fraunhofer Institute for Molecular Biology and Applied Ecology (IME), Branch Translational Medicine and Pharmacology, 60596 Frankfurt, Germany

We thank Fabbris et al. for their remarks [1] on our publication “Self-Collected Samples to Detect SARS-CoV-2: Direct Comparison of Saliva, Tongue Swab, Nasal Swab, Chewed Cotton Pads and Gargle Lavage” [2].

We agree that nasal wash or nasopharyngeal aspirate, which has previously been demonstrated to be useful when testing for different viruses, Ref. [3] may also be an interesting candidate to test for SARS-CoV-2 in a self-collected environment.

In our study, we limited the number of different collection techniques to avoid overwhelming the study participants with a multitude of samples collected without supervision by a medical professional, and due to potential interference between different specimens.

Our study was designed to assess the diagnostic sensitivity of self-collected specimens. Therefore, we recruited patients who were known to be infected with SARS-CoV-2. This prohibited us from determining the specificity of the materials examined in our study.

## References

[1] Fabbris C., Camerotto R., Battistuzzi V., Spinato G. (2022). A Viable Alternative. Comment on Kohmer et al. Self-Collected Samples to Detect SARS-CoV-2: Direct Comparison of Saliva, Tongue Swab, Nasal Swab, Chewed Cotton Pads and Gargle Lavage. *J. Clin. Med.* 2021, *10*, 5751. J. Clin. Med..

[2] Kohmer N., Eckermann L., Böddinghaus B., Götsch U., Berger A., Herrmann E., Kortenbusch M., Tinnemann P., Gottschalk R., Hoehl S. (2021). Self-Collected Samples to Detect SARS-CoV-2: Direct Comparison of Saliva, Tongue Swab, Nasal Swab, Chewed Cotton Pads and Gargle Lavage. J. Clin. Med..

[3] Flynn M.F., Kelly M., Dooley J.S.G. (2021). Nasopharyngeal Swabs vs. Nasal Aspirates for Respiratory Virus Detection: A Systematic Review. Pathogens.

